# Trans-anal Minimally Invasive Surgery Combined With a Robotic Anterior Approach for Sleeve Resection of a Huge Rectal Gastrointestinal Stromal Tumor

**DOI:** 10.7759/cureus.46288

**Published:** 2023-09-30

**Authors:** Daibo Kojima, Takahisa Fujikawa, Ryuji Kajitani, Yoshiko Matsumoto, Suguru Hasegawa

**Affiliations:** 1 Gastroenterological Surgery, Fukuoka University Hospital, Fukuoka, JPN; 2 Surgery, Kokura Memorial Hospital, Kitakyushu, JPN

**Keywords:** rectourethral muscle, imatinib mesylate, robotic rectal surgery, trans-anal minimally invasive surgery, rectal gastrointestinal stromal tumor

## Abstract

Due to anatomical complexity, large rectal gastrointestinal stromal tumors (GISTs) in the pelvis at the anterior aspect often require extended abdominal surgery to obtain clear surgical margins. Here, we show our trans-anal minimally invasive surgery combined with a robotic anterior approach for a huge low rectal GIST that was widely in contact with the prostate and urethra. By performing lateral dissection first, we can identify the orientation of critical organs such as the prostate, urethra, and neurovascular bundles, facilitating anterior anorectal dissection without urethral injury. Although the combination with a transabdominal robotic approach was required because of firm inflammatory adhesion between the tumor and prostate, the preceding trans-anal dissection plane facilitated the robotic anterior dissection and contributed to achieving complete dissection with negative resection margins.

## Introduction

Gastrointestinal stromal tumors (GISTs) are the most common mesenchymal neoplasms of the gastrointestinal tract and originate from interstitial cells of Cajal, which are known as gut pacemaker cells. GISTs rarely occur in the low rectum, which accounts for <1% of the total [1]. Large rectal GISTs in the pelvis often require extended abdominal surgery to obtain clear surgical margins, even though the ideal would be local excision keeping the tumor capsule intact while preserving the anal sphincter and urogenital function. The optimal surgical approach depends on multiple factors including tumor size, location, and presence of local invasion [2]. Recently, the efficiency of trans-anal minimally invasive surgery in rectal cancer has been demonstrated, but challenges remain for achieving safe dissection of the anterior anorectum without margin involvement [3]. We have reported a safe and simple technique for anterior dissection in trans-perineal abdominoperineal resection using a simultaneous laparoscopic and trans-perineal endoscopic approach [4,5]. In this report, we present trans-anal minimally invasive surgery combined with a robotic anterior approach for a huge low rectal GIST with neoadjuvant imatinib mesylate therapy.

## Technical report

**Figure 1:**
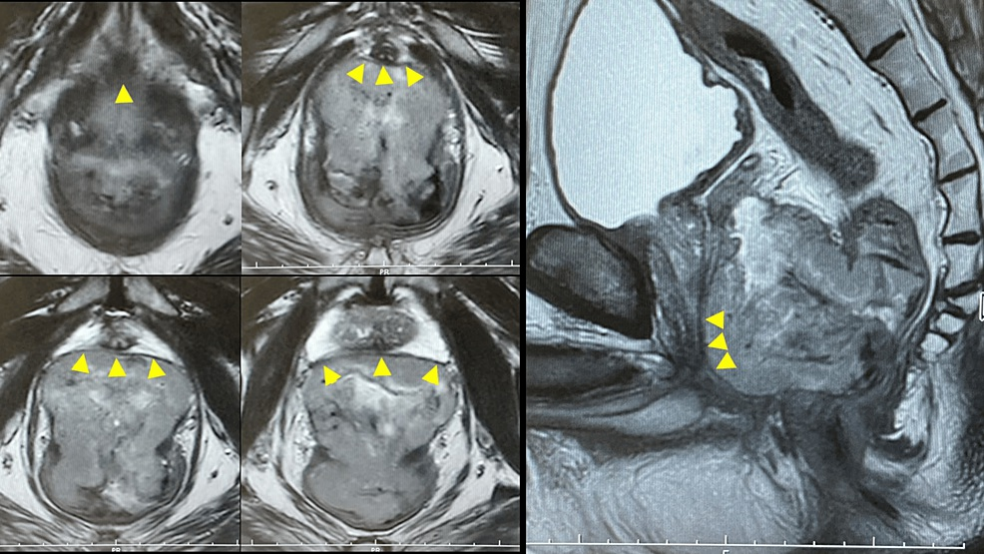
MRI findings prior to the imatinib treatment The MRI showed that a huge rectal GIST was widely in contact with the prostate and urethra (yellow arrows).

The patient was a 66-year-old man with a huge low rectal GIST at the anterior aspect that measured 70 mm and was widely in contact with the prostate and urethra (Figure 1). No distant metastasis was detected on computed tomography, so he underwent neoadjuvant imatinib mesylate therapy, expecting tumor shrinkage.

**Figure 2:**
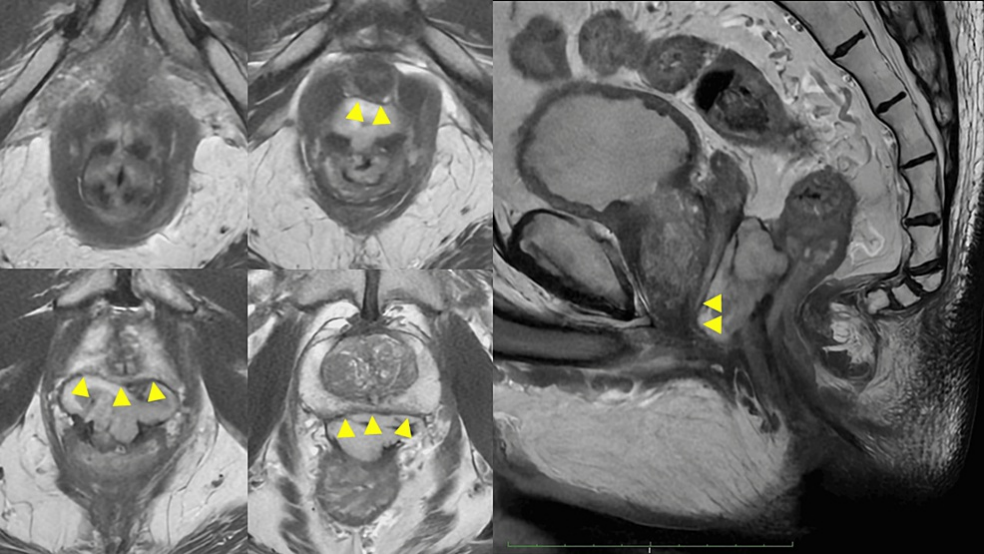
MRI findings after the imatinib treatment The MRI showed that the tumor shrunk to 45 mm, but was still in extensive contact with the prostate and urethra (yellow arrows).

After 12 months of imatinib mesylate therapy, the tumor had shrunk remarkably to 45 mm, but it remained in extensive contact with the prostate and urethra, though without any signs of tumor invasion (Figure 2). Thus, we planned complete local excision of the tumor by trans-anal minimally invasive surgery.

Strategy

**Figure 3:**
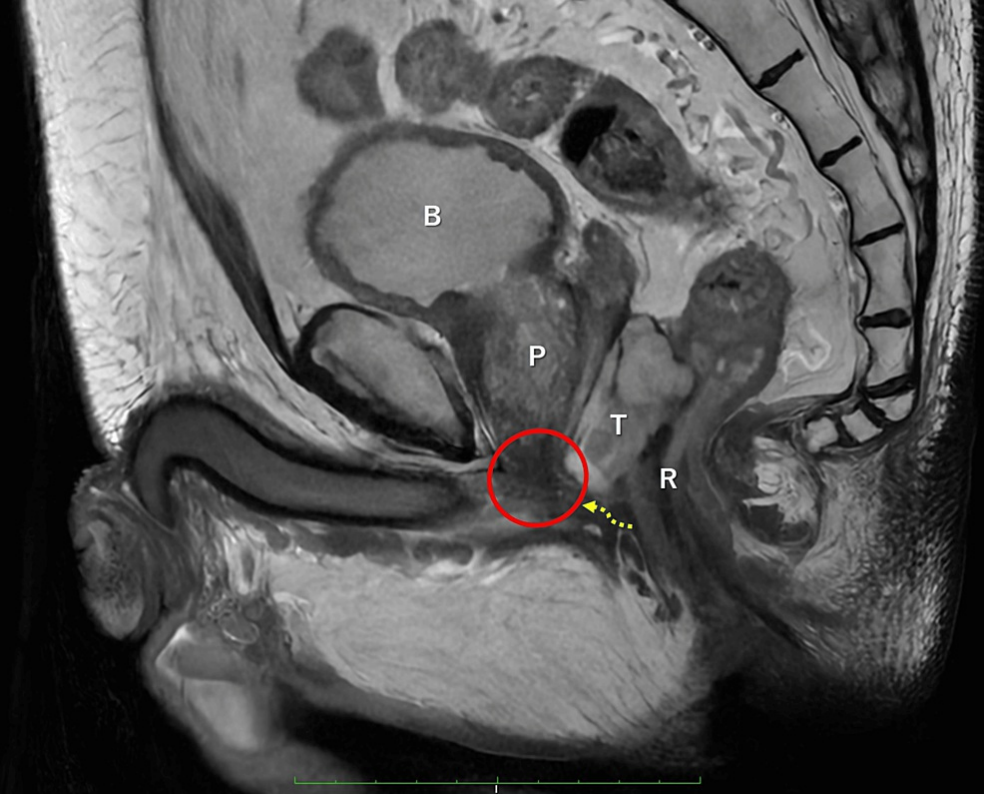
Strategy for surgery, avoiding urethral injury On sagittal MRI, the caudal end of the tumor was located at the level of the urethra, where the dissection line was obscured by the complex anatomy, particularly, the rectourethral muscle (red circle). Therefore, immediate dissection of this area would have increased the risk of straying into the urethra or tumor. B, bladder; P, prostate; T, tumor; R, rectum

On sagittal MRI, the caudal end of the tumor was located at the level of the urethra, where the dissection line was obscured by the complex anatomy, particularly, the rectourethral muscle (Figure 3). Therefore, immediate dissection of this area would have increased the risk of straying into the urethra or tumor.

**Figure 4:**
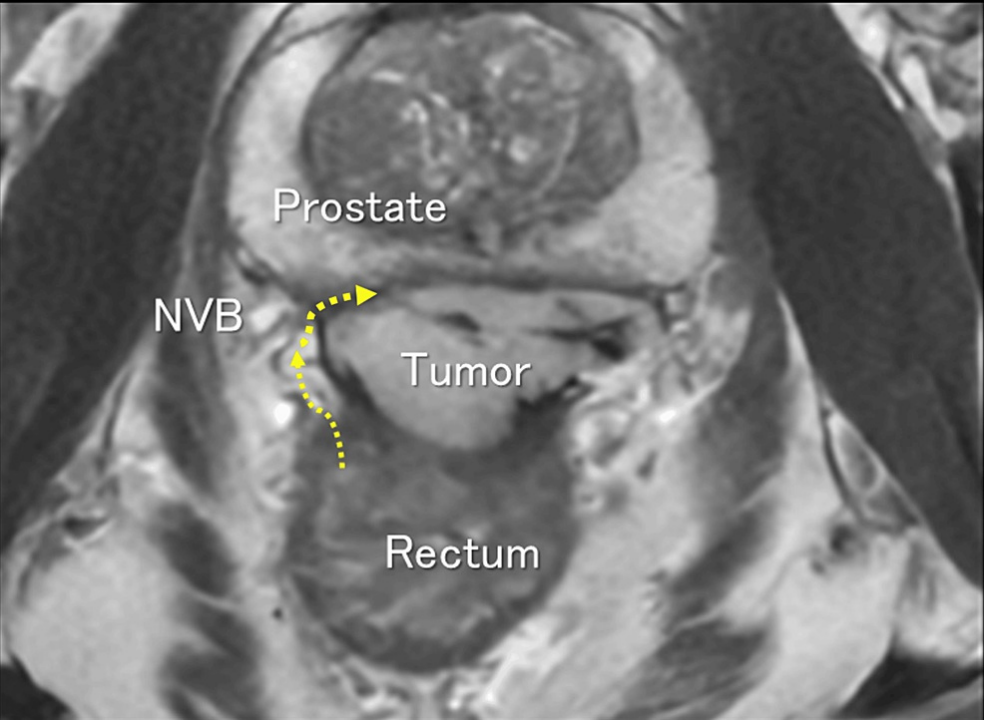
Strategy for surgery, identifying the prostate, the key extraluminal landmark On axial MRI, we planned to start with lateral dissection and identify the surface of prostate, the key extraluminal landmark (yellow arrow). NVB, neurovascular bundles

We planned to start with lateral dissection and identify the surface of the prostate, the key extraluminal landmark (Figure 4). After dissecting the full thickness of the rectal side wall and mesorectum, taking care to avoid injury to the tumor capsule and neurovascular bundles, the dissection can reach near the prostate surface. By performing lateral dissection first, we can identify the orientation of critical organs such as the prostate, urethra, and neurovascular bundles, facilitating anterior anorectal dissection without urethral injury. Electrically stimulated contraction of the puborectalis muscle also helped to identify the anterior dissection plane.

Operative procedure

**Figure 5:**
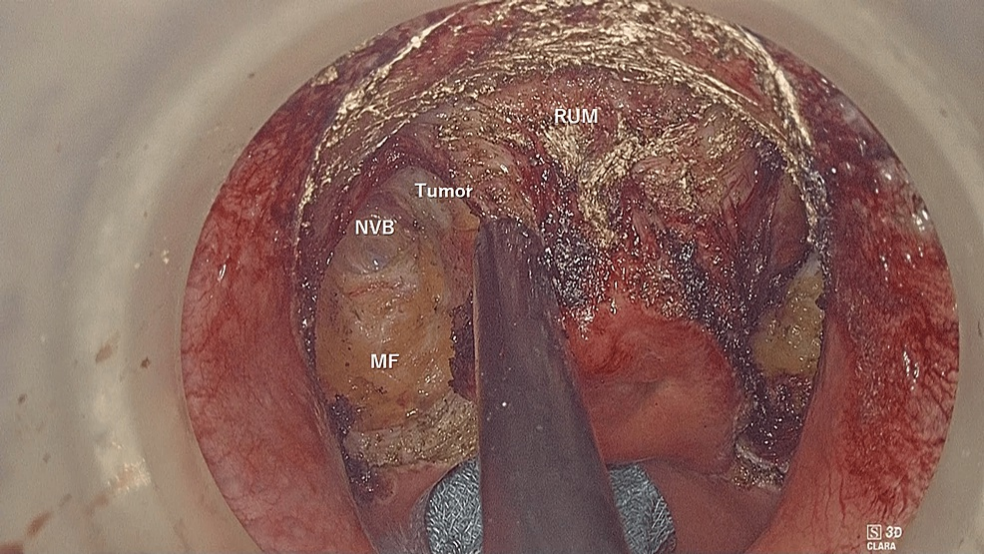
Surgical view after bilateral dissection RUM, rectourethral muscle; NVB, neurovascular bundles; MF, mesorectal fat

Following circumferential marking of the mucosal incision line, we first made a full-thickness incision dividing the rectum on both sides of the tumor. Then, the mesorectum was divided, and neurovascular bundles and the surface of the prostate, the key anatomical landmark in this operation, were identified (Figure 5).

**Figure 6:**
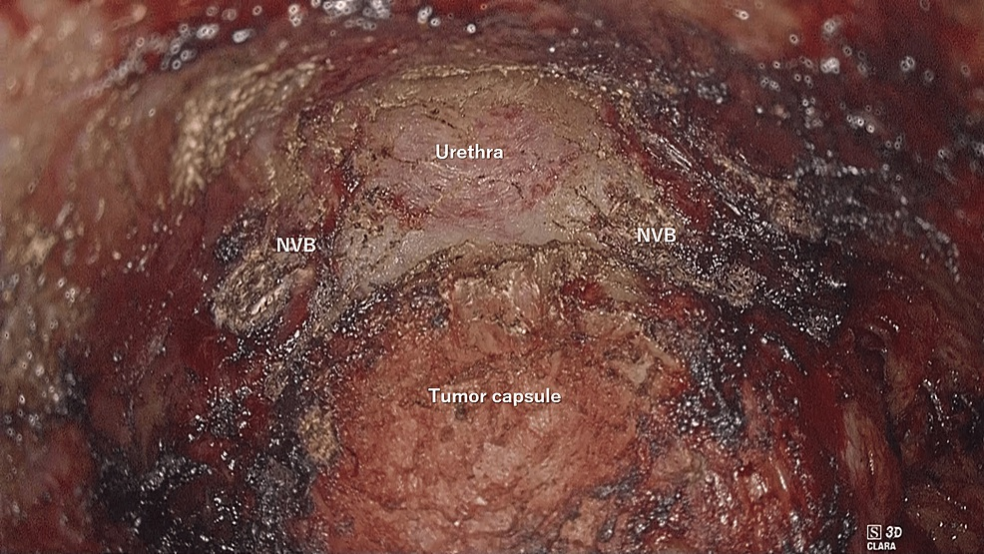
Surgical view of safe anterior anorectal dissection Safe dissection of the anterior anorectum was done and the urethra was identified while keeping the tumor capsule intact. NVB, neurovascular bundles

Next, we moved on to anterior-caudal dissection. Anteriorly, the complex anatomy made identification of the dissection line difficult, but prior identification of the prostate helped us identify it just outside of the tumor. Traction of a urinary catheter helped to identify the prostate. In this way, safe dissection of the anterior anorectum was achieved while keeping the tumor capsule intact (Figure 6).

Although surgery combined with a transabdominal robotic approach was required because of firm inflammatory adhesion between the tumor and prostate probably due to effects of neoadjuvant therapy, the preceding trans-anal dissection plane facilitated the robotic dissection and contributed to achieving complete dissection with negative resection margins.

Histopathological findings

Final histopathological findings revealed a GIST measuring 42 mm that was positive for DOG-1 and C-kit, with Ki-67 index 23% and a mitotic index of 5 per 80.

Video 1 shows the strategy, operative procedure and histopathological findings of this case.

**Video 1:** Trans-anal minimally invasive surgery combined with a robotic anterior approach for sleeve resection of a huge rectal gastrointestinal tumor

Outcomes

The better trans-anal visualization and preceding trans-anal dissection contributed to achieving complete dissection with negative resection margins. There were no intraoperative complications. At the 18-month follow-up, the anal sphincter and urogenital function were well preserved with no recurrence.

## Discussion

Huge rectal GISTs are a challenge even for the specialists of colorectal surgery. As rectal GISTs do not metastasize to lymph nodes, anterior resection has little benefit. Trans-anal excision is inadequate for large, extra-rectal growing tumors and abdominoperineal resection is overtreatment for the tumor with intact anal canal. So, in this case, we planned complete local excision using trans-anal minimally invasive surgery keeping the tumor capsule intact while preserving the anal sphincter and urogenital function.

The optimal surgical approach depends on multiple factors including tumor size, location, and presence of local invasion. Various surgical techniques have been described for rectal GISTs, including conventional trans-anal resection, trans-sacral approach [6], trans-vaginal approach [7], trans-anal endoscopic microsurgery (TEM) [8], trans-anal minimal invasive surgery [9,10], and laparoscopic surgery, though the appropriate surgical technique is still debated.

Because of the difficulty of locating the tumor’s extraluminal component through a trans-anal approach, a trans-sacral approach in the prone position has been reported to obtain better visualization of the anterior surgical field, but this approach involves large skin incisions even when skin is spared with tumor invasion. This approach is also associated with a higher rate of fistula formation [11]. TEM is only indicated for small lesions that have decreased to <2 cm post-imatinib with intact sphincter [8].

Recently, the efficacy of trans-anal endoscopic surgery in rectal cancer surgery has been demonstrated [3-5]. The technical advantages and better visualization that this approach affords may also facilitate better outcomes for patients with locally advanced rectal GISTs, though little is known about the risk and benefits, and there are no indication criteria available. Trans-anal minimally invasive surgery for rectal GISTs has been reported, though they are the intraluminally growing type and located at the posterior aspect, or even anteriorly, and are small in size [9,10]. Trans-anal minimally invasive surgery for rectal GISTs particularly the extraluminal growing type at the anterior aspect is technically challenging due to anatomical complexity. In this case, by performing lateral dissection first, we could identify the orientation of critical organs such as the prostate, urethra, and neurovascular bundles, facilitating anterior anorectal dissection without urethral injury. Electrically stimulated contraction of the puborectalis muscle also helped to identify the anterior dissection plane. The better trans-anal visualization, keeping the surgical field airtight, facilitated the identification of the prostate, neurovascular bundles and urethra, the key extraluminal landmark of this surgery. Safe dissection of the anterior anorectum was achieved while keeping the tumor capsule intact.

The effect of neoadjuvant imatinib mesylate therapy has been reported [12,13], though in this case, firm inflammatory adhesion between the tumor and prostate was seen and combining surgery with a transabdominal robotic approach was required. The preceding trans-anal dissection plane facilitated the robotic dissection and contributed to achieving complete dissection with negative resection margins.

## Conclusions

Trans-anal minimally invasive surgery for a huge rectal GIST at the anterior aspect is safe and feasible. This approach helped us to identify the prostate, urethra, and neurovascular bundles, the important extramural structure, and safe anterior anorectal dissection was achieved without urethral injury or damage to the tumor capsule. Preceding trans-anal dissection can facilitate robotic or laparoscopic transabdominal anterior dissection and may improve oncological and functional outcomes.
